# Spatiotemporal Trends and Co-Resistance Patterns of Multidrug-Resistant Enteric *Escherichia coli* O157 Infections in Humans in the United States

**DOI:** 10.3390/pathogens14090888

**Published:** 2025-09-05

**Authors:** Tarjani Bhatt, Csaba Varga

**Affiliations:** 1 Department of Health and Kinesiology, University of Illinois Urbana-Champaign, Urbana, IL 61802, USA; tkbhatt2@uillinois.edu; 2 Department of Pathobiology, College of Veterinary Medicine, University of Illinois Urbana-Champaign, Urbana, IL 61802, USA; 3 Carl R. Woese Institute for Genomic Biology, University of Illinois Urbana-Champaign, Urbana, IL 61802, USA

**Keywords:** Shiga toxin-producing *Escherichia coli* O157, antimicrobial resistance, multidrug resistance, geographic disparities, temporal trends, public health surveillance

## Abstract

Multidrug-resistant (MDR) Shiga toxin-producing *Escherichia coli* O157 (STEC O157) is a public health threat. This study analyzed publicly available surveillance data collected by the National Antimicrobial Resistance Monitoring System (NARMS) to assess temporal and regional differences and co-resistance patterns in MDR STEC O157 human clinical isolates across the United States. Co-resistance patterns were assessed by hierarchical clustering and Phi coefficient network analyses. A negative binomial regression model estimated the incidence rate ratios (IRRs) for the number of antimicrobial classes to which an isolate was resistant, across years and geographic regions. Out of 1955 isolates, 151 (7.57%) were MDR. The most important clusters were Cluster 1 (*n* = 1632), which included susceptible isolates, and Cluster 3 (*n* = 255), comprising the majority of the MDR isolates, having a high resistance prevalence to tetracyclines (TET) (0.97), folate pathway inhibitors (FPI) (0.77), and phenicols (PHN) (0.49). In the co-resistance network, TET, FPI, and PHN served as central hubs, with large nodes and thick edges, suggesting that they are frequently co-selected. The highest IRRs were observed in Regions 6 (IRR = 2.72) and 9 (IRR = 2.00), compared to Region 4. Compared to 2010, a significant increase in the IRR was observed in each year from 2015 to 2021 (IRRs 2.5–4.38). Antimicrobial stewardship programs and public health interventions targeting MDR *E. coli* O157 are needed to mitigate the emergence of antimicrobial resistance.

## 1. Introduction

Shiga toxin-producing *Escherichia coli* O157 (STEC O157) is an important enteric pathogen transmitted to humans via contaminated food products or direct contact with animals in North America and worldwide [1,2]. STEC O157 cause infections ranging from mild diarrhea to more severe symptoms, including hemorrhagic colitis and hemolytic uremic syndrome (HUS) [3]. Globally, STEC infections are estimated to cause approximately 2.8 million illnesses annually [1]. In the United States of America (US), the Centers for Disease Control and Prevention (CDC) estimates that STEC O157 causes 97,000 illnesses, 3270 hospitalizations, and 30 deaths annually, with high hospitalization and mortality rates among vulnerable populations, including children and immunocompromised individuals [4].

In the US, STEC O157 is present in beef cattle and might pose a risk to food safety if beef products enter the food chain or if humans are exposed to infected animals [5]. Moreover, outbreaks linked to consumption of STEC O157-contaminated undercooked beef, pork products, poultry, leafy greens, and raw dairy products are well documented [6,7,8,9]. Also, regional and temporal differences in STEC O157 infections have been described, suggesting the impact of local livestock farm densities and demographic determinants of the local population [10,11].

The emergence of multidrug-resistant (MDR) STEC O157 poses a public health threat as it decreases treatment success for severe infections [12]. While antibiotic therapy is not recommended for STEC O157 infections due to the risks of increasing Shiga toxin release and triggering HUS, in cases with bacteriemia or severe co-infections, antimicrobial therapy should be considered [13,14]. Monitoring MDR STEC O157 isolates is important as these strains could cause extraintestinal infections and also serve as a reservoir for antimicrobial resistance genes. A recent surveillance study from Michigan, US, described a high prevalence of antimicrobial resistance (AMR) in STEC O157 isolated from clinical cases with diarrhea [15]. Also, in England, the emergence of STEC O157 strains was observed, and they were frequently resistant to aminoglycosides, tetracyclines, and sulphonamides, and less frequently to fluoroquinolones, macrolides, and third-generation cephalosporins. In addition, several of these isolates were MDR, and their resistance was encoded by antimicrobial resistance genes co-located on mobile genetic elements [16].

In the US, antimicrobial resistance (AMR) surveillance in enteric bacteria is important to identify emerging multidrug-resistant strains, assess the effectiveness of current treatment protocols, and guide antibiotic stewardship [17]. The National Antimicrobial Resistance Monitoring System (NARMS) is a collaborative program established in 1996 in the US, a partnership among the Centers for Disease Control and Prevention (CDC), the U.S. Food and Drug Administration (FDA), and the U.S. Department of Agriculture (USDA), along with state and local health departments to track AMR in foodborne bacteria of humans (overseen by CDC), retail meat (overseen by FDA), and food animals at slaughter establishments (overseen by USDA) [18]. The CDC leads the AMR monitoring of enteric bacteria, including STEC O157, that are isolated from human sporadic and outbreak cases [18]. Human clinical STEC O157 isolates are tested for resistance to a panel of antimicrobials representing major drug classes to detect emerging resistance trends. The data generated are shared publicly via dashboards and annual reports to inform the general public, policy, outbreak response, and antimicrobial stewardship efforts [19]. However, these reports use descriptive statistics and describe temporal and regional trends for individual antimicrobials, and do not provide information on co-resistance patterns and emerging multidrug resistance determinants.

Our study aims to address this gap by employing two unsupervised machine learning approaches, a hierarchical single-linkage clustering with the Jaccard similarity coefficient, and a network analysis with the Phi coefficient, to assess co-resistance patterns in STEC O157 clinical isolates of humans. Additionally, a negative binomial regression model will assess the impact of time and location on the number of antimicrobial classes to which an STEC O157 isolate is resistant.

We hypothesize that multidrug resistance in STEC O157 is determined by co-resistance to certain antimicrobial classes. We also hypothesize that MDR STEC O157 will exhibit significant temporal and regional variations, with certain geographic areas and periods showing higher resistance to multiple classes of antimicrobials. To test these hypotheses, publicly available data on AMR in STEC O157 clinical isolates of humans collected by the CDC NARMS across the US between 2010 and 2021 were analyzed.

Our study provides information on multidrug resistance patterns and identifies regional and temporal factors influencing multidrug resistance in STEC O157 isolates of humans that are not evident from existing publicly available reports and dashboards. The ultimate goal is to inform antimicrobial stewardship policies and programs to mitigate the emergence of multidrug resistance in clinical STEC O157 isolates.

## 2. Materials and Methods

### 2.1. Study Design and Data Source

Publicly available data on AMR in clinical human enteric STEC O157 isolates between 2010 and 2021 were obtained from the CDC NARMS program [19]. Public health laboratories in all 50 states across the US and Washington, D.C., send every 20th STEC O157 isolate from sporadic cases of illness, and all isolates linked to multistate outbreaks, regardless of the 1-in-20 sampling protocol, to the CDC NARMS laboratory for antimicrobial susceptibility testing [20].

The CDC reports regional antimicrobial resistance data using the U.S. Department of Health and Human Services (HHS) regional framework, which divides the US into ten administrative regions. These regions are composed as follows: Region 1: Connecticut, Maine, Massachusetts, New Hampshire, Rhode Island, Vermont; Region 2: New Jersey, New York, Puerto Rico, Virgin Islands; Region 3: Delaware, District of Columbia, Maryland, Pennsylvania, Virginia, West Virginia; Region 4: Alabama, Florida, Georgia, Kentucky, Mississippi, North Carolina, South Carolina, Tennessee; Region 5: Illinois, Indiana, Michigan, Minnesota, Ohio, Wisconsin; Region 6: Arkansas, Louisiana, New Mexico, Oklahoma, Texas; Region 7: Iowa, Kansas, Missouri, Nebraska; Region 8: Colorado, Montana, North Dakota, South Dakota, Utah, Wyoming; Region 9: Arizona, California, Hawaii, Nevada, Pacific Islands; and Region 10: Alaska, Idaho, Oregon, Washington [21].

### 2.2. Antimicrobial Susceptibility

The CDC NARMS laboratory uses the broth microdilution method to define the minimum inhibitory concentrations (MICs) for each isolate against the following antimicrobial classes: aminoglycosides (GEN: gentamicin), β-lactam combination agents (AUG: amoxicillin-clavulanic acid), cephems (AXO: ceftriaxone; FOX: cefoxitin), folate pathway inhibitors (COT: trimethoprim-sulfamethoxazole; FIS: sulfisoxazole), penicillins (AMP: ampicillin), phenicols (CHL: chloramphenicol), quinolones (CIP: ciprofloxacin, NAL: nalidixic acid), tetracyclines (TET: tetracycline), and macrolides (AZI: azithromycin). Resistance to antimicrobial agents was interpreted based on Clinical and Laboratory Standards Institute (CLSI) breakpoints [22].

### 2.3. Statistical Analyses

#### 2.3.1. Descriptive Statistics

All statistical analyses were conducted using R statistical software (Version 4.5.1 (2025-06-13)) (R Core Team, 2020) within the RStudio platform (R Studio Version 1.4.1106© 2009–2021 RStudio, PBC). ArcGIS Pro version 3.0.3 (Environmental Systems Research Institute, Inc. (ESRI), Redlands, CA, USA) was used to construct maps.

For resistance classification, intermediate isolates were included in the resistant category. Multidrug resistance (MDR) was defined as resistance to three or more antimicrobial classes [23]. The prevalence of MDR by year and region was calculated with exact binomial 95% confidence intervals and was illustrated in a figure and a map.

#### 2.3.2. Regression Analysis

A multivariable negative binomial regression model [24] was fitted to evaluate the association between year- and region-independent variables, and the dependent variable representing the number of antimicrobial classes to which an STEC O157 isolate was resistant. The year categorical independent variable included the year 2010 as the referent, while the region categorical variable included the region with the lowest average resistance as the referent, respectively. Incidence rate ratios (IRRs) and 95% confidence intervals were computed by exponentiating the model coefficients and illustrated in figures. A *p*-value ≤ 0.05 was considered significant. Compared to the referent category, an IRR of <1 indicated a decrease, and >1 an increase in the rate of MDR STEC O157 isolates. All regression analyses were conducted in R (version 4.3.1) using the ‘MASS’, ‘dplyr’, and ‘broom’ packages.

#### 2.3.3. Assessing Co-Resistance Patterns

##### Clustering Analysis

A similarity cluster analysis was conducted on STEC O157 isolates based on their resistance (0 = susceptible, 1 = resistant) to eight antimicrobial classes: aminoglycosides (AGs), β–lactam combination agents (BLIs), cephalosporins (CEPs), folate pathway inhibitors (FPIs), penicillins (PENs), phenicols (PHNs), quinolones (QNs), and tetracyclines (TETs). The binary resistance matrix was used to compute pairwise Jaccard distances among isolates using the ’proxy’ R package (version 2.1.4). Hierarchical clustering was applied to the resulting matrix, and clustered heatmaps were generated using the ‘ComplexHeatmap’ R package [25]. Cluster membership was assigned using a 4-cluster solution, and proportions of resistance within each cluster were summarized across all drug classes.

##### Network Analysis

A network analysis for co-resistance patterns was completed for the previously described eight antimicrobial classes. The Phi coefficient, a measure of association between two binary variables, resistance (yes/no) to antimicrobial classes, was used to quantify pairwise co-resistance. A threshold of ≥0.01 was used to identify all co-resistance relationships. The network was visualized using a circular layout with edge thickness proportional to the Phi coefficient and node size reflecting the prevalence of resistance for each antimicrobial class. Network centrality metrics, including degree, betweenness, and closeness, were calculated and illustrated in a figure using the igraph package in R [26].

## 3. Results

### 3.1. Prevalence of MDR E. coli O157 by Year and Region

**Figure 1:**
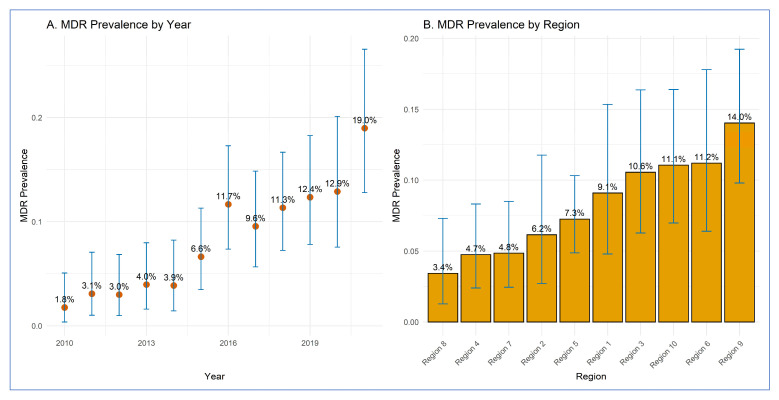
Distribution of multidrug-resistant *E. coli* O157 isolates from human clinical cases by (**A**) year and (**B**) region. (**A**) Orange dots represent the yearly proportion (%) of MDR isolates, with vertical blue lines showing the 95% confidence intervals calculated using the exact binomial method. (**B**) Boxes represent the MDR proportion (%) for each region, with the box height corresponding to the proportion level. Vertical blue lines show the 95% confidence intervals, calculated using the exact binomial method.Geographic variation in MDR STEC O157 prevalence across the ten regions was observed (Figure 1B and Figure 2).

The annual and regional proportions of MDR STEC O157 clinical human isolates are presented in Table 1 and Figure 1.

**Table 1:** Distribution of multidrug-resistant Shiga-toxin producing *E. coli* O157 recovered from human clinical infections in the United States by year and region.

Variable	MDR Isolates	Total Isolates	Proportion (%)	95% CI
**Year**				
2010	3	170	1.76	0.4–5.1
2011	5	162	3.09	1.0–7.1
2012	5	167	2.99	1.0–6.8
2013	7	177	3.95	1.6–8.0
2014	6	155	3.87	1.4–8.2
2015	12	181	6.63	3.5–11.3
2016	21	180	11.67	7.4–17.3
2017	17	178	9.55	5.7–14.9
2018	22	194	11.34	7.2–16.7
2019	21	170	12.35	7.8–18.3
2020	16	124	12.90	7.6–20.1
2021	26	137	18.98	12.8–26.6
**Region**				
Region 1	12	132	9.10	4.8–15.3
Region 2	8	130	6.20	2.7–11.8
Region 3	17	161	10.60	6.3–16.4
Region 4	11	232	4.70	2.4–8.3
Region 5	28	386	7.30	4.9–10.3
Region 6	15	134	11.20	6.4–17.8
Region 7	11	227	4.80	2.4–8.5
Region 8	6	175	3.40	1.3–7.3
Region 9	32	228	14.00	9.8–19.2
Region 10	21	190	11.10	7.0–16.4

**Figure 2:**
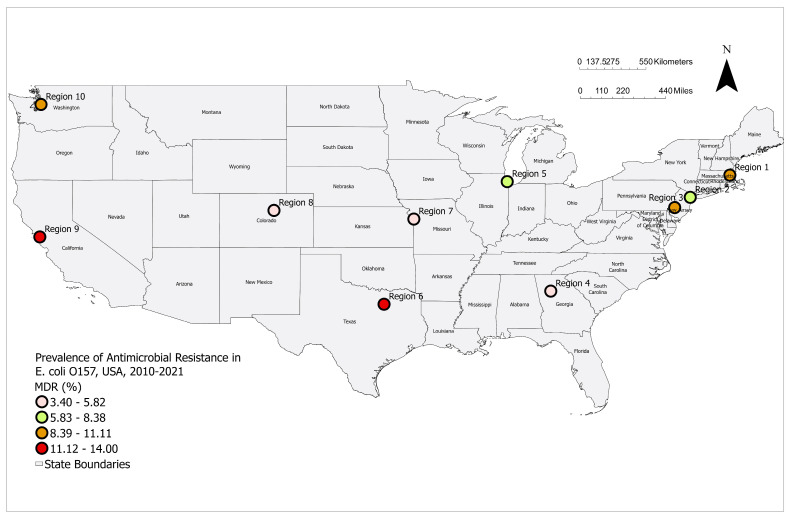
Distribution of multidrug-resistant Shiga-toxin producing *E. coli* O157 clinical isolates in humans across regions of the US.

Between 2010 and 2021, the proportion of MDR STEC O157 clinical isolates in humans increased. In 2010, 1.8% (95% CI: 0.4–5.1%) of isolates were MDR (3 out of 170). This proportion rose gradually in the following years, reaching 6.6% (95% CI: 3.5–11.3%) in 2015 and 12.4% (95% CI: 7.8–18.3%) in 2019. The highest annual prevalence was observed in 2021, with 19.0% (95% CI: 12.8–26.6%) of isolates identified as MDR (26 out of 137) (Figure 1).

Regions 9 and 6 reported the highest MDR proportion, followed by Regions 1, 3, and 10. In contrast, Regions 4, 7, and 8 reported the lowest MDR prevalence.

### 3.2. Regression Analysis Results

**Figure 3:**
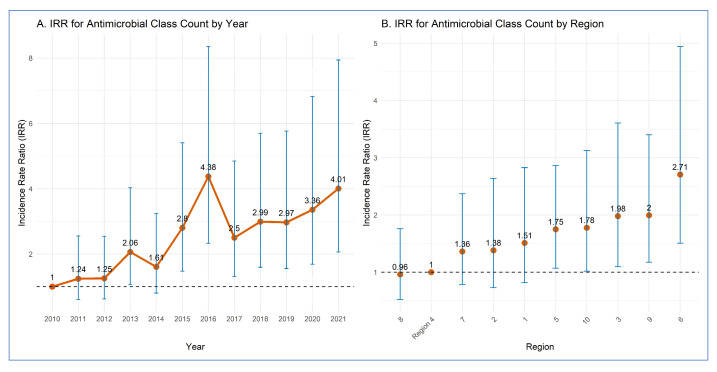
Results of a multivariable negative binomial regression model on the associations between the incidence of multidrug-resistant *E. coli* O157 clinical isolates across (**A**) years and (**B**) regions. (**A**) Yellow dots represent the estimated incidence rate ratios (IRR) for each subsequent year (2011–2021), with vertical lines denoting their 95% confidence intervals. The thicker yellow line connects the yearly IRR estimates to illustrate temporal trends relative to the baseline year. A horizontal dotted line represents the reference (IRR = 1), indicating no difference compared to 2010; yearly IRR estimates whose 95% confidence intervals overlap this line are not statistically significant. (**B**) Yellow dots represent the IRR for each region, and the vertical blue lines denote their 95% confidence intervals, with Region 4 as the reference (IRR = 1). The horizontal dotted line at IRR = 1 indicates the reference, indicating no difference compared to Region 4, and IRR estimates whose 95% confidence intervals overlap this line are not statistically significant.

Table 2 and Figure 3 display the results of the negative binomial regression model that assessed the associations between the dependent variable representing the number of antimicrobial classes to which an STEC O157 isolate was resistant, and two independent categorical variables, year and region.

Compared to the reference year of 2010, significantly higher rates of resistance to antimicrobial classes were observed in 2013, 2015, 2016, 2017, 2018, 2019, 2020, and 2021. In other years, no statistically significant differences were observed relative to 2010.

Regarding regional differences, isolates from Region 6 had the significantly highest rate of resistance to antimicrobial classes (IRR = 2.71) compared to the reference region (Region 4), which had the lowest rate. Other regions (Region 9, 3, 10, and 5) had significantly higher rates (1.75–2.00) compared to Region 4. The other regions did not differ significantly from Region 4.

### 3.3. Co-Resistance Patterns

#### 3.3.1. Cluster Analysis Results

A Jaccard similarity cluster analysis was performed on STEC O157 clinical isolates from human enteric cases to identify groups of isolates with similar resistance profiles. Four distinct clusters were identified based on resistance to a panel of antimicrobial classes, including aminoglycosides (AGs), β-lactam combination agents (BLIs), cephalosporins (CEPs), folate pathway inhibitors (FPIs), penicillins (PENs), phenicols (PHNs), quinolones (QNs), and tetracyclines (TETs).

**Figure 4:**
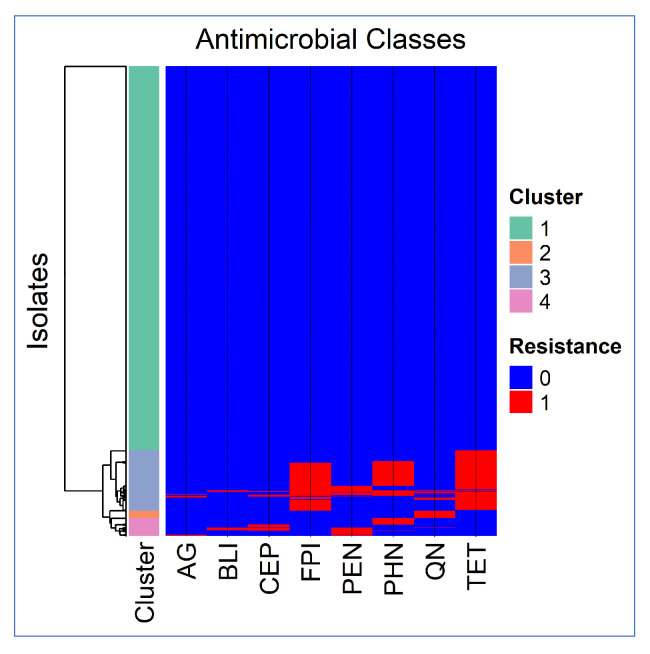
Clustered heatmap of Jaccard similarity among *E. coli* O157 isolates based on their resistance to different antimicrobial classes. Rows represent isolates, columns represent antimicrobial classes. Color indicates resistance status: red = resistant, blue = susceptible. Row-side bars indicate cluster membership. Aminoglycosides (AGs), β-lactam combination agents (BLIs), cephalosporins (CEPs), folate pathway inhibitors (FPIs), penicillins (PENs), phenicols (PHNs), quinolones (QNs), and tetracyclines (TETs).

The cluster analysis heatmap is presented in Figure 4.

Among the 1995 STEC O157 isolates. Cluster 1 consisted of the majority of isolates (1632 isolates, 81.8%), which were susceptible to all the tested antimicrobial classes. Cluster 2 was a smaller group (31 isolates, 1.5%) with resistance only to quinolones. Cluster 3 was larger (255 isolates, 12.8%) and included the majority of the MDR STEC O157 isolates, having a high resistance prevalence to tetracyclines (0.97), folate pathway inhibitors (0.77), and phenicols (0.49), and a lower prevalence of resistance to other antimicrobial classes, including penicillins (0.16) quinolones (0.09), cephalosporins (0.05), aminoglycosides (0.03) and β-lactam combination agents (0.03). Cluster 4 (77 isolates, 3.9%) showed a varied resistance profile with moderate resistance to penicillins (0.44), phenicols (0.39), cephalosporins (0.32), and β-lactam combination agents (0.14), while resistance to aminoglycosides (0.09) and quinolones (0.01) was lower, and for folate pathway inhibitors (0) and tetracyclines (0) was absent.

#### 3.3.2. Network Analysis Results

Table 3 summarizes the network metrics and prevalence values for each antimicrobial class in STEC O157 isolates.

The network degree represents the number of direct co-resistance links, with a higher degree signifying a more co-occurring resistance pattern. In this network, AGs, BLIs, FPIs, PENs, PHNs, and TETs all have a maximum degree of 7, indicating they are highly interconnected in the resistance network. On the other hand, CEPs and QNs have a degree of 6, and they are slightly less involved in co-resistance.

The betweenness reflects how often a particular antimicrobial class serves as a bridge in the shortest paths between other antimicrobial classes. A higher betweenness value means the antimicrobial plays a key role in linking different clusters of AMR. In this network, the majority of antimicrobial classes (AGs, BLIs, FPIs, PENs, PHNs, and TETs) have an identical value of 0.166, suggesting that they all have an equal role in linking different AMR groups. In contrast, CEPs and QNs have a betweenness of 0, indicating that they are not serving as significant bridges in the network.

The closeness represents the inverse of the total shortest path distance to all other nodes, with a higher value signifying the node is more central in the network. In this network, AGs, BLIs, FPIs, PENs, PHNs, and TETs have a closeness value of 0.1429, indicating they are central in the network. On the other hand, CEPs and QNs have a lower value of 0.1250, suggesting they are more peripheral compared to the other antimicrobial classes.

The prevalence represents the proportion of isolates resistant to an antimicrobial class, with a higher value signifying a higher burden. In this network, TETs have the highest prevalence at 0.1238, followed by FPIs at 0.0987. The other antimicrobial classes, AGs, BLIs, CEPs, and QNs, have lower prevalences, suggesting these antimicrobials are less commonly associated with resistance in the isolates.

Considering all of these network metrics, TETs show the highest prevalence and full centrality in the network, indicating they are both widespread and highly connected. FPIs and PHNs are also central and moderately prevalent, suggesting they play important roles in multidrug resistance. On the other hand, CEPs and QNs were less central and prevalent, suggesting a peripheral role in multidrug resistance in STEC O157 isolates.

**Figure 5:**
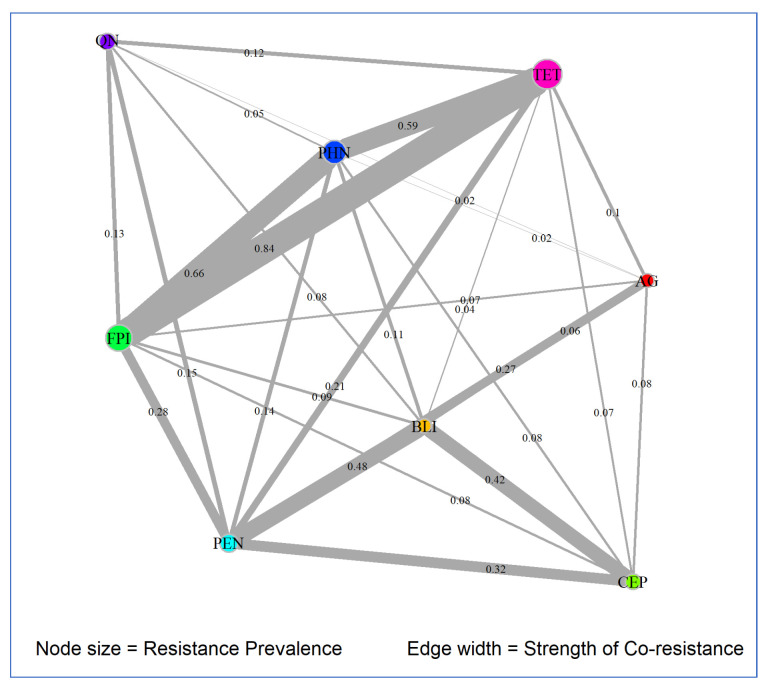
Circular co-resistance network of resistance to various antimicrobial classes in *E. coli* O157 clinical isolates of humans. Nodes (circles) represent resistance to individual antimicrobials, with node size proportional to the prevalence of resistance. Edges (lines) connect antimicrobials that co-occur, with thickness proportional to the strength of association measured by the Phi coefficient.In this network, edges represent co-resistance relationships between antimicrobial classes, with edge thickness proportional to the strength of the relationship, as indicated by the Phi coefficient. In addition, the node size corresponds to the prevalence of resistance for each antimicrobial class, with larger nodes representing higher resistance prevalence. In this network, TETs, FPIs, and PHNs were central nodes, with large node sizes showing their high resistance prevalence, and thick edges signifying their high Phi coefficients, suggesting that they are frequently co-selected in the same STEC O157 isolates. These antimicrobial classes were also interconnected with several other antimicrobial classes, suggesting they are an important part of multidrug resistance patterns due to co-selection of resistance. A second co-resistance network included PENs, BLIs, and CEPs, with slightly smaller node sizes and edge thicknesses than those in the other network; however, this network is also important and suggests that the resistance to these antimicrobial classes is co-selected in the same clinical STEC O157 isolates. In contrast, QNs were positioned at the periphery of the network, with a smaller node, weaker edge thickness, and fewer connections, indicating lower prevalence and less frequent co-occurrence with other antimicrobial resistance classes.

A circular co-resistance network was constructed to visualize the resistance pattern network of various antimicrobial classes in STEC O157 clinical isolates from human cases (Figure 5).

## 4. Discussion

This study analyzed publicly available data on AMR in human enteric clinical STEC O157 isolates (*N* = 1995), assessing temporal and regional differences, and their multidrug resistance patterns. The analysis revealed both temporal and regional variation in multidrug resistance among human STEC O157 isolates. Compared to 2010, the start of the study period, an increase in the incidence rate ratio of multidrug resistance was observed starting in 2015, which continued until 2021. The highest burden of MDR STEC O157 isolates was observed in Regions 6 and 9, suggesting geographic disparities that might relate to differences in local antimicrobial stewardship, population health practices, or transmission dynamics.

The majority of STEC O157 isolates (*n* = 1632; 81.8%) were susceptible to all the tested antimicrobials; however, a cluster of MDR STEC O157 isolates (*n* = 255; 12.8%) was identified that was resistant to multiple antimicrobial classes, including a high resistance prevalence to tetracyclines, folate pathway inhibitors, and phenicols. In addition, the network analysis identified that resistance to tetracyclines, folate pathway inhibitors, and phenicols acted as central nodes, having high prevalence (large node sizes) and high Phi bivariate correlation coefficients (thick edges), suggesting that they are frequently co-selected in the same STEC O157 isolates.

Recent studies have identified significant regional and temporal differences in the antimicrobial resistance profiles of enteric commensal and pathogenic *Escherichia coli* isolates of livestock, humans, and the environment [27]. As STEC O157 isolates can be transmitted to humans via the food chain or contact with animals, the emergence of antimicrobial resistance in any segment of the food chain poses a human health risk which warrants a One Health approach to mitigate it [28]. A current study from the US evaluated the data collected by NARMS, assessing antimicrobial resistance patterns in commensal *E. coli* isolated from swine cecal samples at slaughter, and found a high prevalence of resistance to tetracycline (67.6%) and an increase in multidrug resistance [29]. In addition, differences in antimicrobial resistance in *E. coli* isolates from different swine production types were demonstrated, identifying market hogs as having higher β-lactam antimicrobial resistance rates compared to sows [30].

Although antimicrobial therapy is not recommended for enteric STEC O157 infections due to the risk of toxin release and triggering HUS. Treatment is necessary in severe or systemic infections, bacteremia, immunocompromised patients, or when extraintestinal dissemination of the bacteria occurs [31]. In such cases, antimicrobial therapy should be guided by antimicrobial susceptibility profiles of STEC O157 isolates, avoiding antimicrobials to which the isolate is resistant [32].

The emergence of resistance to multiple antimicrobials in STEC O157 isolates is a public health issue because it increases morbidity, mortality, treatment failures, and medical costs [33]. Co-resistance against tetracyclines, folate pathway inhibitors (e.g., trimethoprim-sulfamethoxazole), and phenicols (e.g., chloramphenicol) in STEC O157 isolates has been described previously. Several studies have demonstrated that genes conferring resistance to these antimicrobials were located on mobile genetic elements or chromosomes, promoting the spread of resistance across bacterial populations [31]. A study from England assessed antimicrobial resistance in enteric STEC isolated from symptomatic human cases and identified the most common AMR profile, resistance to ampicillin, streptomycin, trimethoprim/sulphonamide, and tetracycline [34], and suggested that transmission of resistant isolates to humans via the food chain or direct contact with animals might be a risk factor for the presence of MDR isolates in humans. Another study from England assessed the AMR surveillance data between 2016 and 2020 in human clinical isolates and identified that the most common resistance determinants conferred resistance to aminoglycosides, tetracyclines, and sulphonamides, while resistance to fluoroquinolones, macrolides, and third-generation cephalosporins was rare [16].

Despite the majority of STEC O157 isolates in our study being susceptible to all tested antimicrobials, these isolates still caused enteric infections. This finding might suggest that antimicrobial resistance is not a prerequisite for pathogenicity. Instead, the presence of specific virulence factors, such as Shiga toxin genes (stx1, stx2), intimin (eae), and hemolysins, plays an important role in causing enteric infections [35]. On the other hand, a previous whole-genome comparative analysis of STEC O157 isolates described that certain virulence and resistance genes were colocated on mobile genetic elements, conferring virulence and antimicrobial resistance to these isolates [36].

For STEC O157 antimicrobial resistance monitoring, state public health laboratories routinely submit every 20th isolate from sporadic cases to CDC NARMS, along with all isolates associated with multistate outbreaks, regardless of the 1-in-20 rule. This approach strengthens national surveillance and emphasizes the importance of multistate outbreak-related strains. However, if MDR STEC O157 strains are more frequently associated with multistate outbreaks, it might overestimate the MDR prevalence in the dataset.

In our study, unsupervised machine learning algorithms, the phi correlation matrix, and Jaccard similarity hierarchical clustering were used to group STEC O157 isolates with similar resistance patterns. This technique is suited to binary resistance data, as it quantifies shared resistance traits without being affected by co-susceptibility. The identified clusters visualized in a heatmap described overall resistance patterns in STEC O157 isolates that might reflect underlying genetic or epidemiological factors impacting co-resistance. This approach has also been used previously to investigate correlations between antimicrobial resistance phenotypes and genotypes in carbapenem-resistant *Escherichia coli* isolates from freshwater aquaculture environments [37].

A new approach has been described by a recent study in Spain that used network analysis to evaluate associations between virulence factor genes and antimicrobial resistance in *Staphylococcus hyicus* isolates from Spanish swine farms [38]. Similarly, our study utilized a Phi coefficient network analysis to evaluate and illustrate, in a graph, the antimicrobial resistance network in enteric STEC O157 isolated from human clinical cases across the US. This approach is useful for understanding the co-resistance patterns among different antimicrobial agents by quantifying the strength and direction of the association among resistance patterns. Also, this approach identifies antimicrobials as central nodes and bridges that are essential to form the AMR network. Moreover, AMR networks can identify clusters of antimicrobials that are co-resistant in the same STEC O157 isolate, suggesting genetic linkages, cross-resistance, or shared environmental pressures. Information on the co-resistance network patterns in STEC O157 isolates can aid health professionals in their therapeutic decision-making by suggesting effective antimicrobials to avoid treatment failures. Lastly, data on local resistance profiles of STEC O157 isolates can inform policy recommendations to mitigate the spread of resistant strains and to develop targeted interventions, including monitoring and controlling the use of specific antimicrobial classes in agriculture, healthcare, and veterinary sectors.

As our study identified regional differences in the rate of MDR STEC O157 isolates, future studies should evaluate local risk factors contributing to these differences, including evaluating environmental, agricultural, livestock density, antimicrobial use patterns, and socio-economic factors that influence the prevalence and distribution of MDR STEC O157 strains. Also, to explore the genetic basis for the co-resistance observed to tetracyclines, folate pathway inhibitors, and phenicols, a whole-genome sequencing analysis of STEC O157 isolates is needed to identify specific resistance genes or plasmids involved in the co-selection of resistance to these antimicrobials.

## 5. Conclusions

This study identified an increase in the rate of MDR STEC O157 isolates starting in 2015 and continuing to the end of the study in 2021. In addition, regional differences in the rate of MDR STEC O157 have been identified, suggesting local risk factors for the selection of MDR strains. The co-resistance heatmap and AMR network identified that resistance to tetracyclines, folate pathway inhibitors, and phenicols co-occurred in the same STEC O157 isolates, implying genetic determinants of resistance or co-selection of resistance. Our study findings highlight the importance of continuous surveillance of AMR in enteric human STEC O157 isolates to identify emerging trends and assess the effectiveness of stewardship programs.

## Figures and Tables

**Table 2:** Results of a multivariable negative binomial regression model on the associations between the incidence of multidrug-resistant *E. coli* O157 clinical isolates across years and regions.

Variable	IRR ^a^	95% CI ^b^ (Lower)	95% CI (Upper)	*p*-Value ^c^
**Year**				
2010 (Referent)	1	-	-	-
2011	1.24	0.61	2.55	0.550
2012	1.25	0.62	2.54	0.531
2013	**2.06**	1.07	4.03	0.033
2014	1.61	0.80	3.24	0.183
2015	**2.80**	1.47	5.41	0.002
2016	**4.38**	2.33	8.35	<0.001
2017	**2.50**	1.31	4.85	0.006
2018	**2.99**	1.59	5.70	0.001
2019	**2.97**	1.55	5.77	0.001
2020	**3.36**	1.68	6.82	0.001
2021	**4.01**	2.06	7.94	<0.001
**Region**				
Region 4 (Referent)	1	-	-	-
Region 1	1.51	0.82	2.83	0.195
Region 10	**1.78**	1.02	3.13	0.044
Region 2	1.38	0.73	2.64	0.326
Region 3	**1.98**	1.10	3.61	0.022
Region 5	**1.75**	1.07	2.86	0.025
Region 6	**2.71**	1.50	4.94	0.001
Region 7	1.36	0.78	2.37	0.272
Region 8	0.96	0.52	1.76	0.896
Region 9	**2.00**	1.17	3.40	0.011
(Intercept)	**0.10**	0.05	0.18	<0.001

^a^ IRR: Incidence rate ratio. Statistically significant values are shown in bold. ^b^ CI: Confidence interval. ^c^ Significant at *p*-value ≤ 0.05.

**Table 3:** Network metrics and prevalence values for each antimicrobial resistance in *E. coli* O157 clinical isolates of humans.

Antimicrobial Class ^1^	Degree	Betweenness	Closeness	Prevalence
AG	7	0.1667	0.1429	0.007
BLI	7	0.1667	0.1429	0.009
CEP	6	0.0	0.125	0.0185
FPI	7	0.1667	0.1429	0.0987
PEN	7	0.1667	0.1429	0.0376
PHN	7	0.1667	0.1429	0.0782
QN	6	0.0	0.125	0.0271
TET	7	0.1667	0.1429	0.1238

^1^ Aminoglycosides (AGs), β-lactam combination agents (BLIs), cephalosporins (CEPs), folate pathway inhibitors (FPIs), penicillins (PENs), phenicols (PHNs), quinolones (QNs), and tetracyclines (TETs).

## Data Availability

The data are publicly available at: https://www.cdc.gov/ncezid/dfwed/beam-dashboard.html, accessed on 28 January 2025.
